# Bouveret Syndrome: A Rare Case and Review of the Literature

**DOI:** 10.7759/cureus.24768

**Published:** 2022-05-06

**Authors:** Spencer Probert, Wenyi Cai, Faiyaz Islam, Nikhil Nanjappa Ballanamada Appaiah, Ali Salih

**Affiliations:** 1 General Surgery, Basildon and Thurrock University Hospital, Mid and South Essex NHS Foundation Trust, Basildon, GBR; 2 Colorectal Surgery, Basildon and Thurrock University Hospital, Mid and South Essex NHS Foundation Trust, Basildon, GBR; 3 Breast Surgery, Basildon and Thurrock University Hospital, Mid and South Essex NHS Foundation Trust, Basildon, GBR

**Keywords:** general surgery, upper gastrointestinal surgery, gallstone disease, choleduodenal fistula, bouveret syndrome, enterotomy, cholelithiasis, gallstone ileus, gastric outlet obstruction, bouveret's syndrome

## Abstract

Bouveret syndrome is a subtype of gallstone ileus, wherein a calculus becomes entrapped in the duodenum via a cholecystocolic fistula, leading to gastric outlet obstruction. Due to the non-specific symptoms the patients present with, a diagnosis is reliant on computed tomography (CT), magnetic resonance imaging (MRI) or direct endoscopic visualisation. We report a case of Bouveret syndrome and review current literature, outlining the aetiopathogenesis and management strategies of this condition.

## Introduction

Bouveret syndrome is a rare form of gallstone ileus, making up 1% to 3% of cases [1]. It commonly presents in elderly women, and it has a high risk of morbidity and mortality. This syndrome proves a diagnostic challenge due to the non-specific signs and symptoms on presentation. Imaging or direct endoscopic visualisation is needed to confirm the diagnosis. The primary goal of the management in these cases is the prompt removal of the obstructing calculus, which can be performed either endoscopically or surgically. Due to the difficulty in diagnosis, clinicians should be aware of this condition and should not hesitate in performing imaging studies in patients known to have cholelithiasis, presenting with features of gastric outlet obstruction.

## Case presentation

A 74-year-old female patient was brought in via ambulance to the Accident and Emergency department. She was confused and was only able to give a basic history. She presented with a history of worsening general malaise over the course of three days, accompanied by features of bowel obstruction, including multiple episodes of vomiting on the day prior to presentation, progressive lower abdominal pain and abdominal bloating. The vomitus was described as dark and foul smelling, with no frank blood or coffee-ground features. She also reported having loose stools which had started after she had taken an over-the-counter laxative.

**Figure 1:**
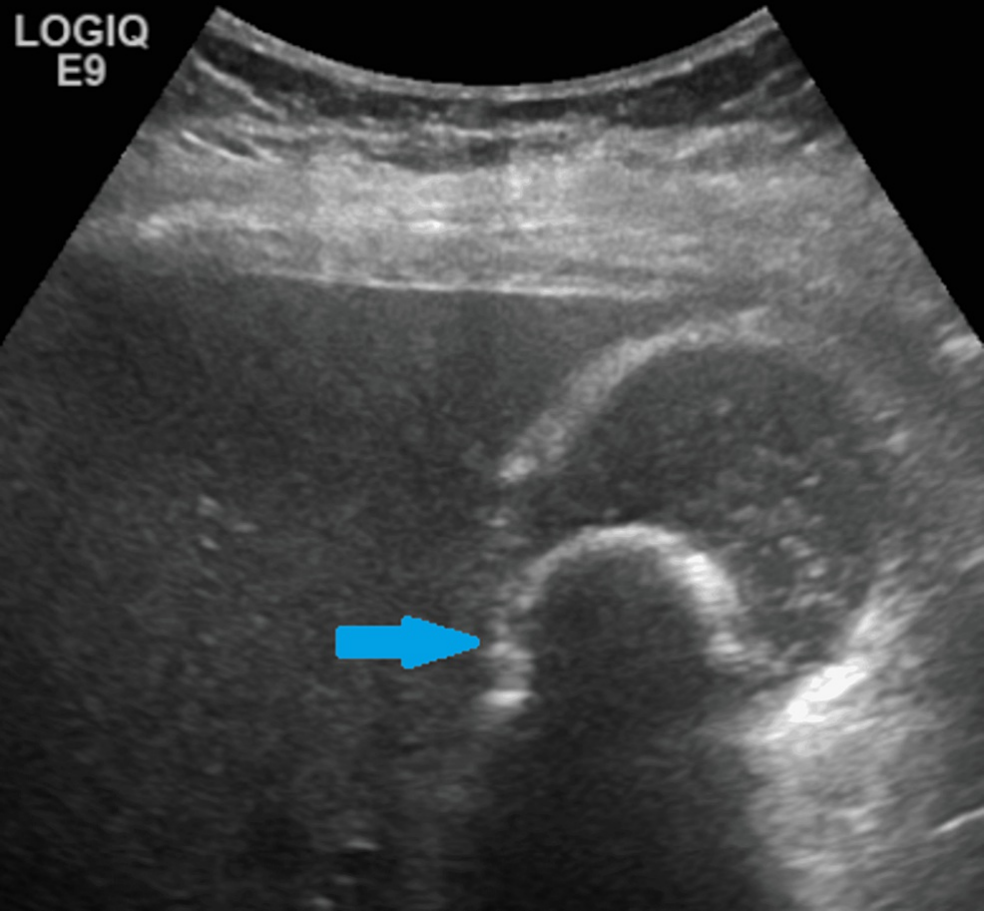
Ultrasound image showing a single 2.7-cm gallstone (blue arrow) within the gallbladder.

The patient has a background of Parkinson's disease, multiple sclerosis (Expanded Disability Status Scale (EDSS) = 7), asthma, non-alcoholic fatty liver disease, depression and cervical spondylosis. These comorbidities are all well controlled on regular medication. Due to multiple sclerosis, the patient is a wheelchair user and is supported at home by her husband. She was diagnosed with cholelithiasis incidentally on an abdominal ultrasound, for investigation of right lower quadrant pain, in August 2020. This noted a large 2.7-cm calculus and echogenic sludge within the gallbladder (Figure 1). The common bile duct (CBD) was of normal calibre with no obvious obstructing calculus. Due to the uncomplicated nature of the calculus, no further follow-up was offered at the time. Following this scan, the patient had remained asymptomatic.

Observations in accident and emergency (A&E) found a tachycardia of 108 beats per minute (bpm), with the remainder being grossly normal. The patient appeared clinically unwell and confused. On abdominal examination, mild right iliac fossa tenderness was noted with fullness, but the abdomen was objectively soft with no signs of peritonitis. Initial blood investigations (Table 1) showed deranged liver function tests with a raised alkaline phosphatase (ALP) and alanine aminotransferase (ALT). The inflammatory markers (both white cell count and C-reactive protein) were also elevated. Due to the history of vomiting and resulting dehydration, both hypokalaemia and raised urea were also found.

**Table 1:** Blood results on initial presentation.

Blood Investigation	Result	Normal Range
Sodium (mmol/L)	144	133-146
Potassium (mmol/L)	3.3	3.5-5.3
Urea (mmol/L)	11.6	2.5-7.8
Creatinine (µmol/L)	76	45-83
Total protein (g/L)	80	60-80
Albumin (g/L)	42	35-50
Globulin (g/L)	38	20-35
Total bilirubin (µmol/L)	17	0-21
Alkaline phosphatase (U/L)	446	30-130
Alanine aminotransferase (U/L)	195	<35
C-reactive protein (mg/L)	89	<5
Amylase (U/L)	24	28-100
Plasma glucose (mmol/L)	10.6	<11.1
Haemoglobin (g/L)	152	115-165
Platelets (x10^9^/L)	322	150-400
White cell count (x10^9^/L)	17.7	4.0-11.0
Differential count (x10^9^/L)		
Neutrophils	15.66	1.7-7.5
Lymphocytes	1.13	1.0-4.5
Monocytes	0.89	0.2-0.8
Eosinophils	0	0.0-0.4
Basophils	0.02	0.0-0.1

**Figure 2:**
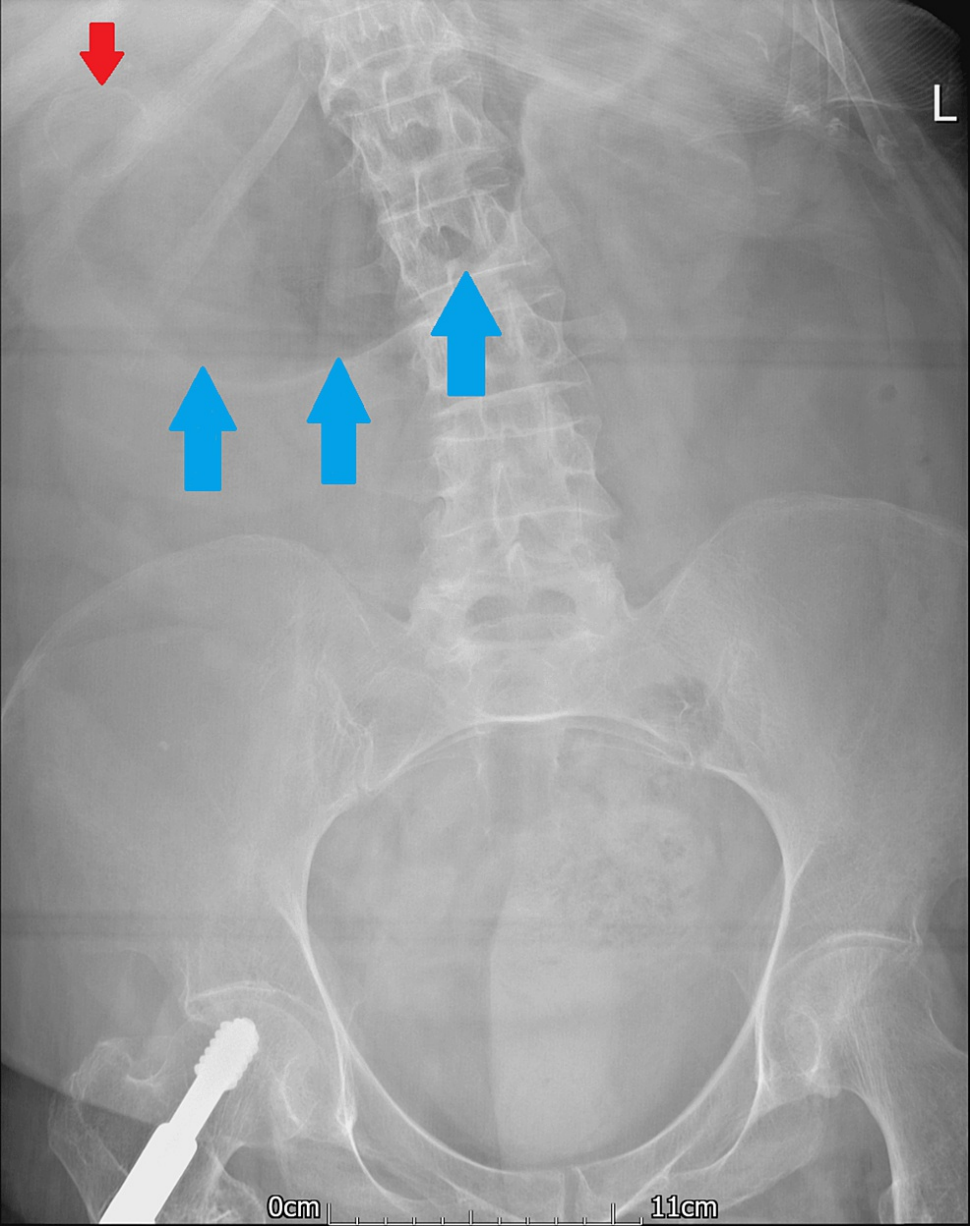
Abdominal x-ray with distended gastric outline (blue arrows). Ring-shaped calcification can be seen in the right upper quadrant of the abdomen (red arrow). L: left.

**Figure 3:**
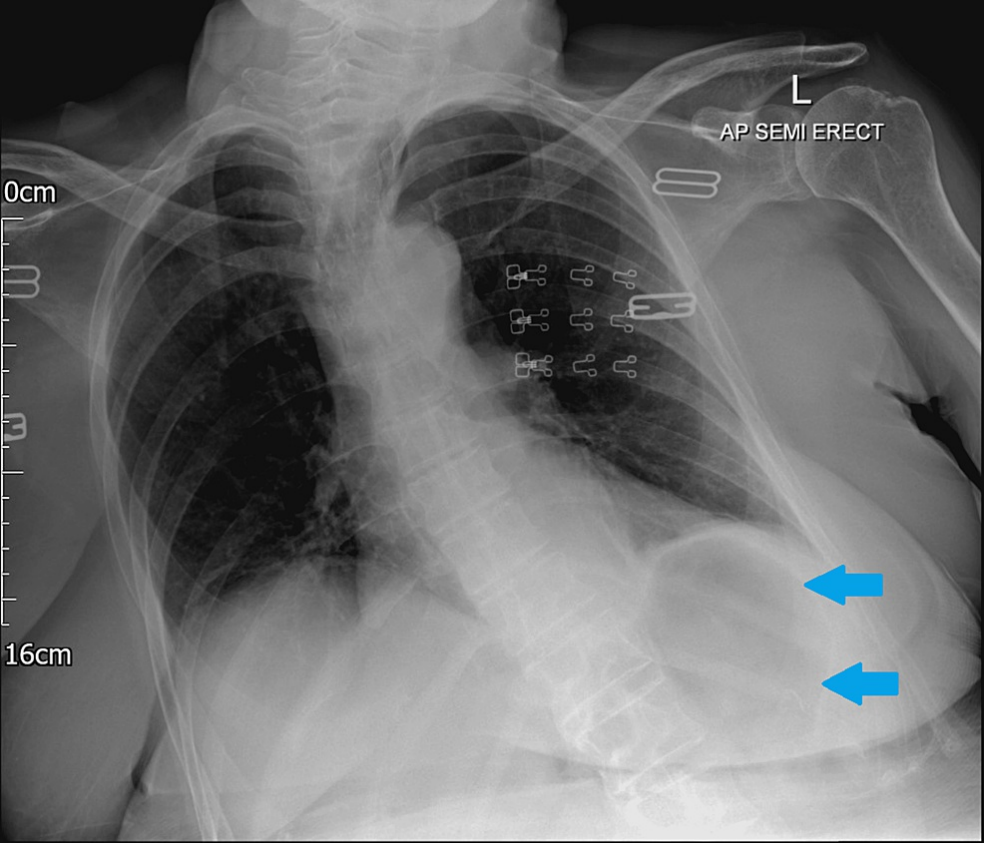
Chest x-ray showing distended gastric outline (blue arrows). L: left, AP: anteroposterior.

**Figure 4:**
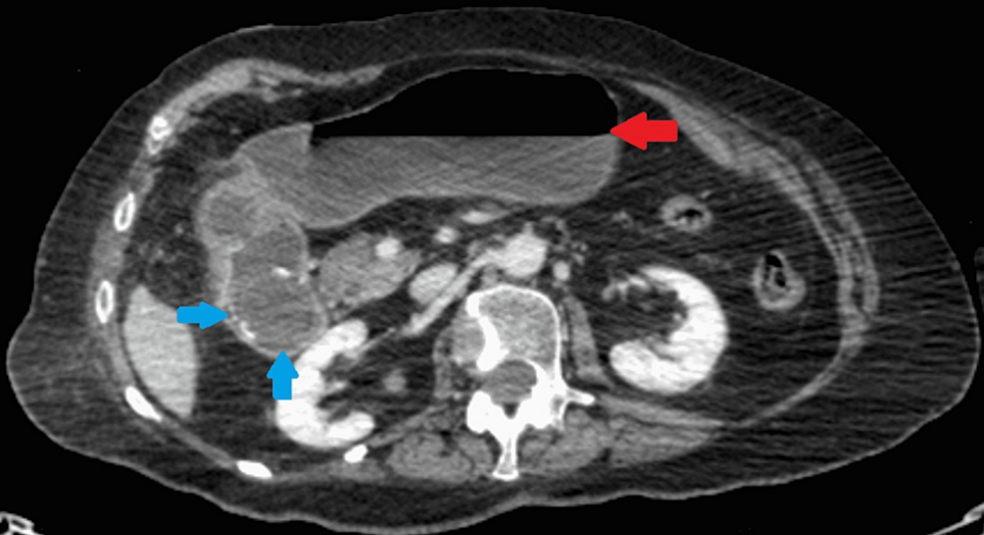
Axial computed tomography image of the abdomen. A calculus (blue arrow) is visualised within the duodenum. Further evidence of gastric dilatation can be seen (red arrow).

**Figure 5:**
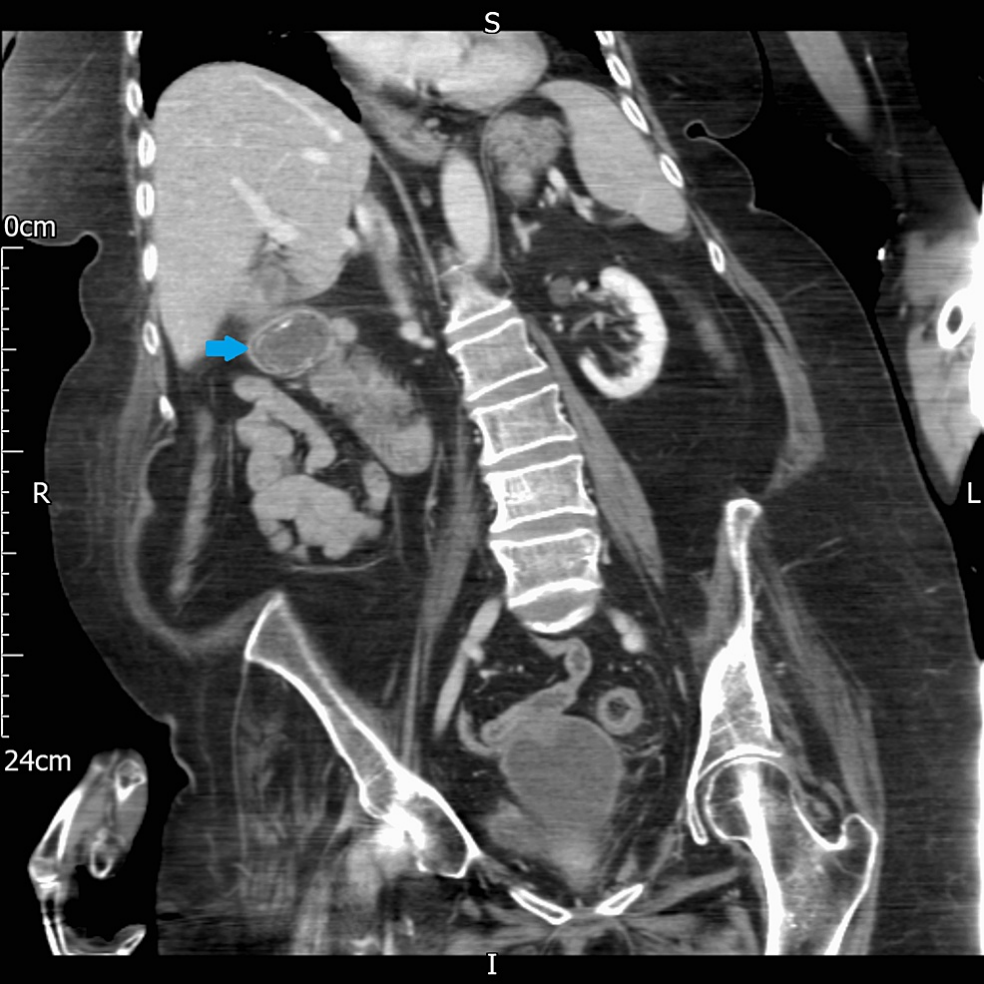
Coronal computed tomography image of the abdomen showing a calculus (blue arrow) within the duodenum. R: right, L: left, S: superior, I: inferior.

**Figure 6:**
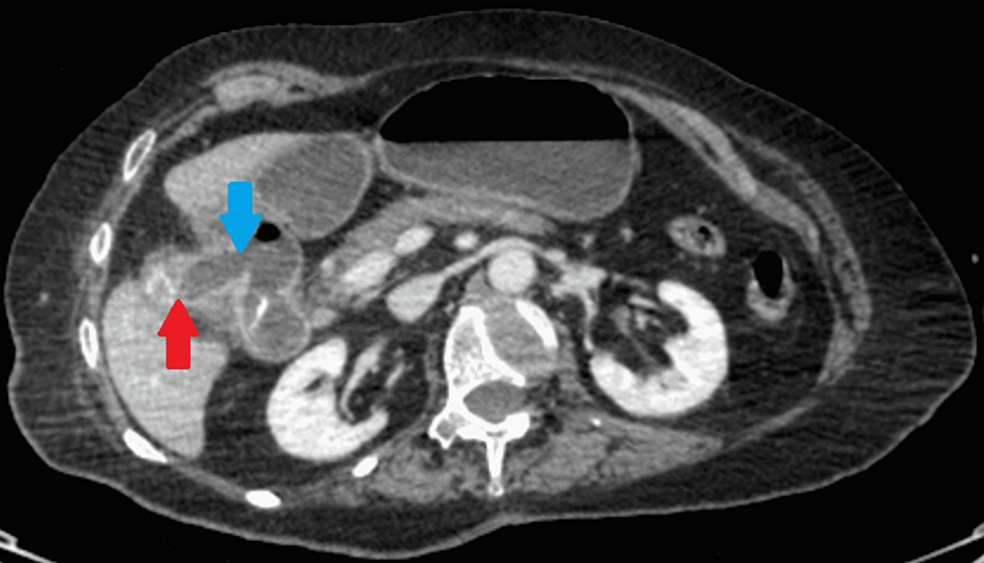
Axial computed tomography image of the abdomen showing a cholecystoduodenal fistula (blue arrow) connecting the gallbladder (red arrow) and duodenum.

**Figure 7:**
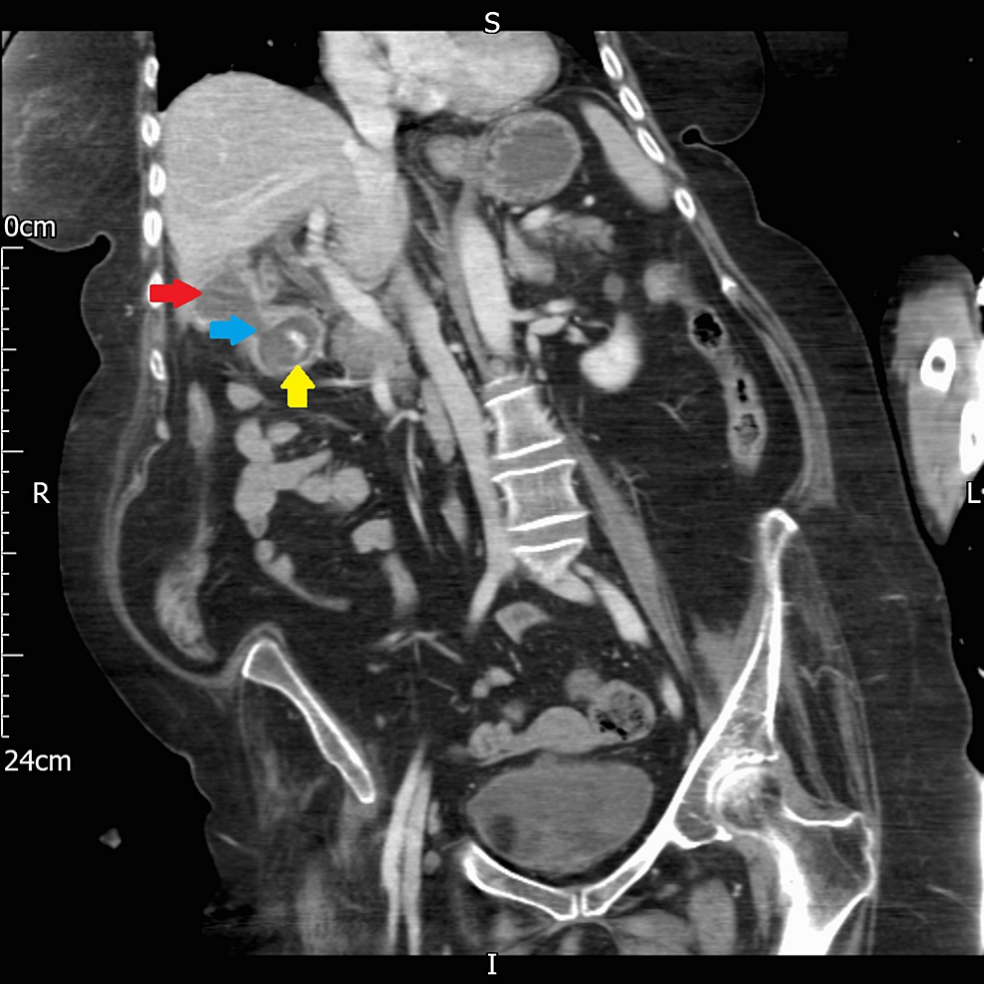
Coronal computed tomography image of the abdomen showing the cholecystoduodenal fistula (blue arrow) connecting the gallbladder (red arrow) and duodenum, wherein there is a large gallstone (yellow arrow). R: right, L: left, S: superior, I: inferior.

**Figure 8:**
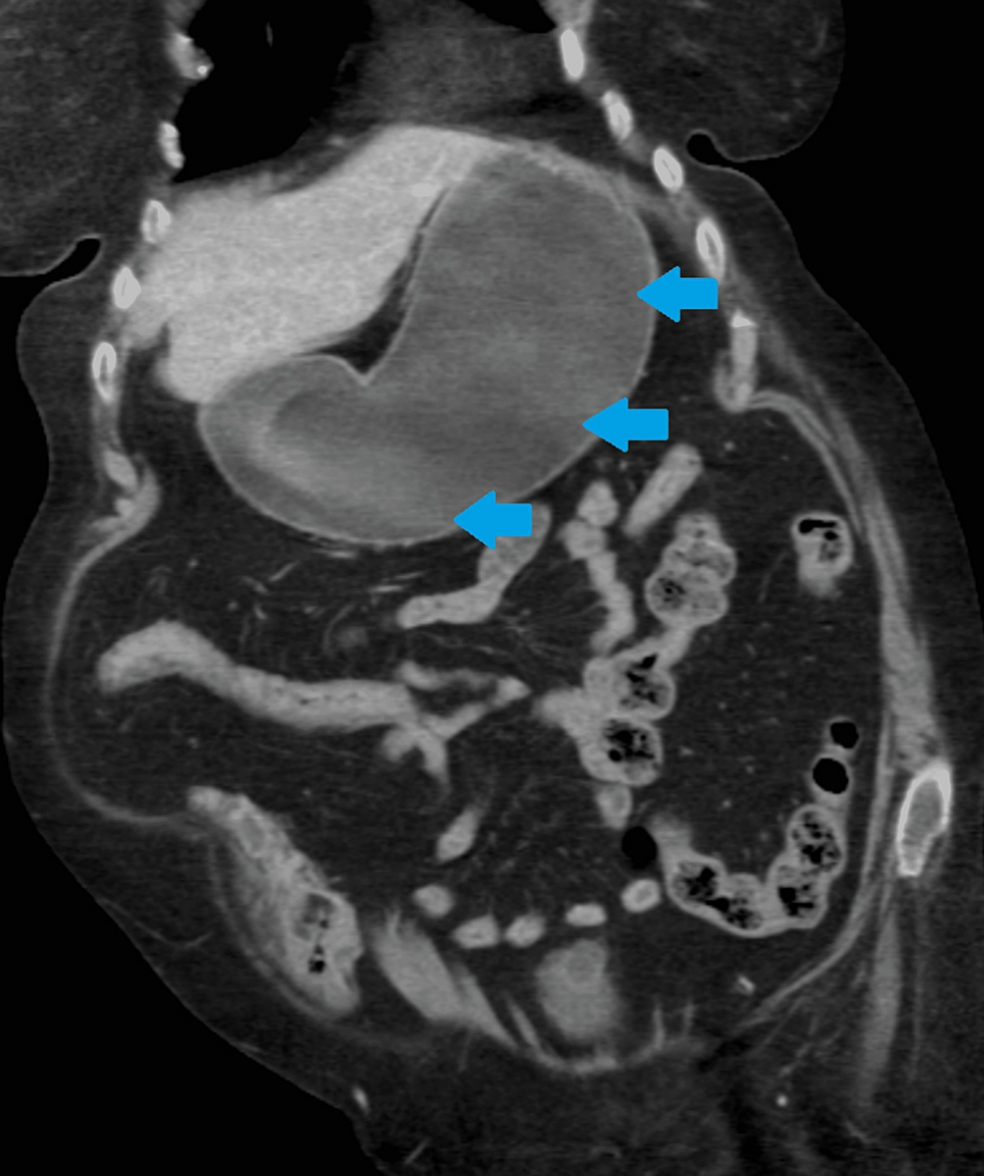
Coronal computed tomography image of the abdomen showing a grossly distended stomach (blue arrows).

**Figure 9:**
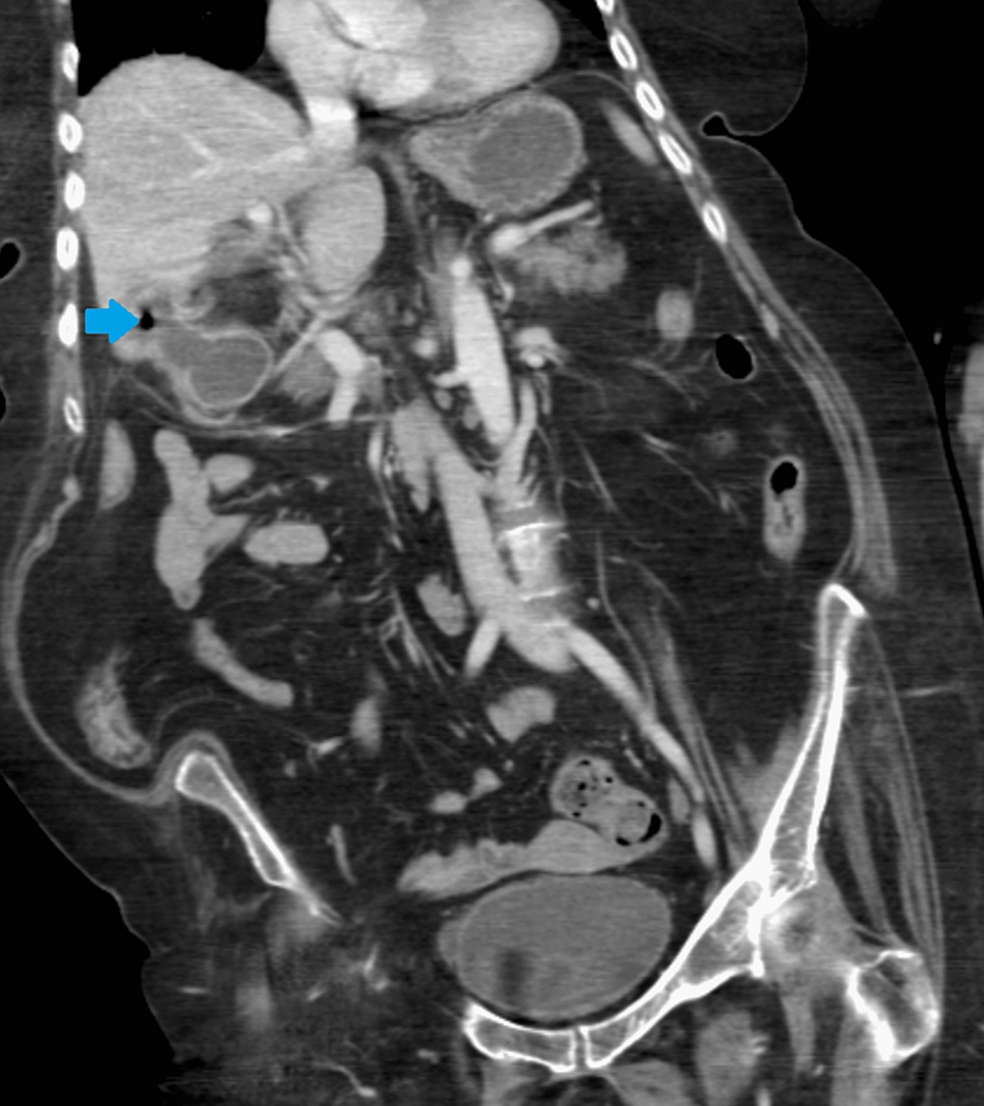
Coronal computed tomography image of the abdomen. Pneumobilia (air within the biliary tree) can be seen (indicated by a blue arrow).

An abdominal and chest x-ray was performed, which showed a dilated stomach (Figures 2, 3) leading to a high clinical suspicion of acute gastrointestinal obstruction. A suspicious ring-shaped calcified structure could also be seen in the right upper quadrant of the abdomen. A CT scan of the abdomen and pelvis was performed to further delineate the pathology. A cholecystoduodenal fistula was revealed arising between the fundus of the gallbladder and the superior duodenal flexure, wherein a partially obstructing calculus was lodged giving rise to upper gastric outlet obstruction (Figures 4-7). Consequently, this led to moderate fluid distension of the stomach and diffuse inflammatory changes in the gallbladder and the hepatoduodenal ligament (Figure 8). Along with the presence of pneumobilia (Figure 9), the radiological findings were all in keeping with Bouveret syndrome. The rest of the small and large bowel was unremarkable in appearance, and there was no free fluid or gas visible in the peritoneal cavity.

**Figure 10:**
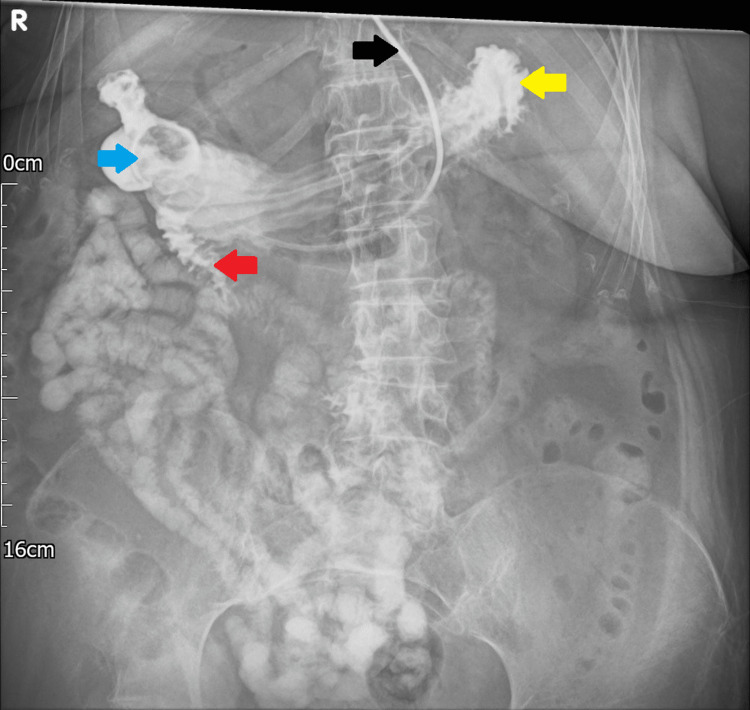
Abdominal x-ray following water-soluble contrast meal. A large obstructing gallstone (blue arrow) can be seen within the duodenum with a small amount of contrast bypassing the obstruction (red arrow). Retention of contrast can be seen within the stomach (yellow arrow). The nasogastric tube, placed for decompression, can be seen within the stomach (black arrow). R: right.

Urgent endoscopic retrieval was attempted. The scope was retroflexed in the stomach and extended distally to the second part of the duodenum. Solid food debris was noted within the stomach and subsequently aspirated through the scope. The duodenum was found to be packed with solid food debris, and jet washing was unable to clear this adequately to establish duodenal anatomy. Thus, the procedure was abandoned. After the failed attempt at endoscopic retrieval, a water-soluble contrast meal using x-ray was performed (Figure 10) to evaluate for any migration of the calculus and to determine any bypass of gastric content past the calculus. This investigation showed no migration and minimal bypass of contrast into the distal small bowel. For definitive surgical management, the patient was transferred to an upper gastrointestinal surgery centre. Whilst awaiting surgical management at this centre, the patient had a peripherally inserted central catheter (PICC) inserted for the administration of total parenteral nutrition (TPN) to provide maintenance of nutrition.

Once transferred to the specialist centre, a repeat endoscopic stone retrieval was attempted which once again was unsuccessful. Following this, the patient underwent a laparoscopic (converted to open) gastrotomy with successful extraction of the gallstone. A triluminal tube (including draining nasogastric and feeding nasojejunal tubes) was inserted for further drainage and administration of nutrition. Postoperatively, the patient was admitted to the intensive care unit for 24 hours following which she was stepped down to the general surgical ward. There were no intraoperative or postoperative complications of note. An intense rehabilitative period ensued which involved physiotherapy, TPN and a gradual reintroduction of oral diet. The patient was admitted for 15 days postoperatively, following which she was discharged having been weaned off of the TPN and tolerating a full oral diet.

## Discussion

Bouveret syndrome, first reported by Leon Bouveret in 1896, is a rare form of gallstone ileus, in which an impacted calculus leads to gastric outlet obstruction via a cholecystoduodenal fistula [2,3]. Bouveret syndrome is most commonly seen in elderly female patients, shown to have a median age of 74 years and a female to male ratio of 1:1.86 [4]. The high rate of morbidity (60%) and mortality (12% to 30%) is attributed to the fact that this syndrome is most prevalent in elderly patients with multiple pre-existing comorbidities and extended diagnostic workup [5]. Risk factors for the development of Bouveret syndrome are similar to those of cholelithiasis. They include a history of cholelithiasis, female gender, age of more than 60 and calculi greater than 2 cm [5].

Bouveret syndrome is thought to be the result of recurrent episodes of cholecystitis, which leads to the development of adhesions between the gallbladder and a neighbouring segment of the upper gastrointestinal tract [6,7]. Pressure necrosis induced by a large calculus leads to the formation of a fistula between the gallbladder and enteric system [5]. The most common type of fistula is cholecystoduodenal flexure; however, cholecystogastric and choledochoduodenal fistulae may also form and lead to the passage of a calculus. Calculi smaller than 2.5 cm often pass through the gastrointestinal tract spontaneously; therefore, calculi leading to Bouveret syndrome are often larger than 2.5 cm in diameter [5]. There is a rare presentation of Bouveret syndrome, in which a patient may have gastric obstructing calculus as well as further calculi which may enter the duodenal lumen, thereby complicating the case with superimposed gallstone ileus [8]. The possibility of this is an important factor to note, as removal of the gastric calculus may not lead to the resolution of the patient's symptoms. This highlights the need for adequate imaging before any procedure is undertaken.

As mentioned, the clinical presentation is often vague, and symptoms are equivalent to that of upper gastrointestinal obstruction. Patients commonly present with vomiting, abdominal pain and reduced appetite. Rarely, these patients may also present with haematemesis due to peptic ulceration or oesophageal trauma from the passage of the calculus in vomitus. Clinical examination usually identifies abdominal tenderness, abdominal distension and features of dehydration [4]. Routine blood investigations are also non-specific and may indicate a degree of CBD obstruction (due to choledocholithiasis), and the result of persistent vomiting would be evidenced by electrolyte disturbances and a raised serum urea.

Rigler’s triad is considered highly suggestive of gallstone ileus [9]. This is a radiological finding characterised by pneumobilia, small bowel obstruction and an ectopic calculus. This triad may also be found in patients with Bouveret syndrome; however, a dilated stomach is more likely to be found than small bowel obstruction. These findings may be detected on ultrasound imaging, but due to the poor penetration of ultrasound through bowel gas, this is a substandard imaging modality in these cases. The imaging modality of choice would be CT, which has a high sensitivity (93%) and specificity (100%) [5]. CT is extremely useful in identifying the level of obstruction, demonstrating the fistula and identifying the degree of surrounding inflammation. Contrast-enhanced CT will aid in the diagnosis and surgical planning for possible bowel ischaemia [10]. All three components of Rigler’s triad may be observed with CT imaging; however, isoattenuating stones are better visualised with magnetic resonance cholangiopancreatography (MRCP). Endoscopy has the potential to be both diagnostic and therapeutic. A dilated stomach is often found, and the impacted stone may be visualised and retrieved. The presence of impacted food material may lead to the failure of this procedure. The success rate of stone retrieval in endoscopy has been reported as low as 10% [2]. Despite this, endoscopy remains the first-line management strategy in Bouveret syndrome. This is due, in part, to the elderly and comorbid nature of the patients, which makes this “least invasive” option the most ideal [2]. Endoscopy may be accompanied with lithotripsy to aid in the resolution of gastric outlet obstruction and to facilitate the removal of the obstructing calculus. Endoscopic laser lithotripsy may be used alone or in conjunction with extracorporeal shockwave lithotripsy (ESWL). Furthermore, intracorporeal electrohydraulic lithotripsy (IEHL) may also be included, alone or in combination with the other methods, as an alternative modality for calculus removal [2]. The disadvantage of endoscopic management is the risk of advancing the calculus distally and causing a distal gallstone ileus.

The surgical management of Bouveret syndrome is divided into three possible surgical strategies [11]. The first of which is a one-stage procedure where an enterolithotomy, cholecystectomy and repair of the cholecystoduodenal are performed simultaneously. Whilst this manages all the acute problems, there is a high risk of mortality associated with this procedure (20%-30%) [2]. The second management strategy is a two-stage procedure, where an enterolithotomy is performed acutely to resolve the obstruction and an interval cholecystectomy is subsequently performed. This allows the patient to recover from the gastric outlet obstruction, and therefore, the cholecystectomy can be performed on an elective basis in a more stable patient. Finally, the enterolithotomy can be performed alone, and this strategy is of particular importance in the more acutely ill patients with multiple comorbidities [5].

The decision to perform either a laparoscopic or open procedure comes down to the experience of the surgeon and available resources at the centre [12]. It is known that laparoscopic procedures provide a quicker recovery time and smaller physiological insult to the patient [11]. This, therefore, would be of benefit to the types of patients who acquire Bouveret syndrome. However, approximately 50% of these laparoscopic procedures have been found to fail and thus require conversion to an open procedure [6].

## Conclusions

Whilst Bouveret syndrome is a rare condition, it remains a serious and potentially fatal complication of gallstone disease. The non-specific presenting symptoms and clinical signs mean that imaging or endoscopic visualisation is a necessity for diagnosis. By reducing the diagnostic delay, a management strategy can be selected to best suit the patient’s comorbid state. Endoscopy is the recommended first-line management, but it has a high failure rate as evidenced by the abandoned endoscopic attempt in this case report. This preference for non-surgical management highlights the care that needs to be taken in managing the acute gastric outlet obstruction whilst simultaneously navigating the comorbidities and clinical state of the patient.
